# NADPH oxidase 4 is dispensable for skin myofibroblast differentiation and wound healing

**DOI:** 10.1016/j.redox.2023.102609

**Published:** 2023-01-13

**Authors:** Aleksandra Malgorzata Siedlar, Tamara Seredenina, Anna Faivre, Yves Cambet, Marie-José Stasia, Dominik André-Lévigne, Marie-Luce Bochaton-Piallat, Brigitte Pittet-Cuénod, Sophie de Seigneux, Karl-Heinz Krause, Ali Modarressi, Vincent Jaquet

**Affiliations:** a Division of Plastic, Reconstructive and Aesthetic Surgery, Geneva University Hospitals, University of Geneva Faculty of Medicine, Geneva, Switzerland; b Department of Pathology and Immunology, Faculty of Medicine, University of Geneva, Geneva, Switzerland; c Department of Cell Physiology and Metabolism, University of Geneva, Geneva, Switzerland; d Service and Laboratory of Nephrology, Department of Internal Medicine Specialties and of Physiology and Metabolism, University and University Hospital of Geneva, Geneva, Switzerland; e READS Unit, Faculty of Medicine, University of Geneva, Geneva, Switzerland; f Université Grenoble Alpes, CEA, CNRS, IBS, F-38044, Grenoble, France

**Keywords:** Wound healing, NADPH oxidase, NOX4, Transforming growth factor β, Mitochondrial uncoupling protein 2, Myofibroblast differentiation, Cellular bioenergetics, TGF-Β, Transforming growth factor beta, NOX4, NADPH oxidase 4, ECM, Extracelullar matrix, ROS, Reactive oxygen species, Α-SMA, Alpha-smooth muscle actin, Ucp2, Uncoupling protein 2, Hddc3, HD domain-containing protein 3, Islr, Immunoglobulin superfamily containing leucine rich repeat, WC, Wound closure, HE, Hematoxylin-eosin, hDFs, Primary human skin fibroblasts from a healthy donor, p22 mut hDFs, Primary human skin fibroblasts from a patient carrying a mutation in CYBA gene, HFF, Human foreskin fibroblasts, EGF, Epithelial growth factor, FGF2, Fibroblasts growth factor 2, EdU, 5-ethynyl-2'-deoxyuridine, RPKM, Reads per kilobase of exon per million reads mapped, FCCP, Carbonyl cyanide-4 (trifluoromethoxy) phenylhydrazone, OCR, Oxygen consumption rate, ECAR, Extracellular acidifiation rate, CAF, Cancer-associated fibroblasts, Nfr2, Nuclear factor erythroid 2-related factor 2, EMT, Epithelial-mesenchymal transition

## Abstract

Differentiation of fibroblasts to myofibroblasts is governed by the transforming growth factor beta (TGF-β) through a mechanism involving redox signaling and generation of reactive oxygen species (ROS). Myofibroblasts synthesize proteins of the extracellular matrix (ECM) and display a contractile phenotype. Myofibroblasts are predominant contributors of wound healing and several pathological states, including fibrotic diseases and cancer. Inhibition of the ROS-generating enzyme NADPH oxidase 4 (NOX4) has been proposed to mitigate fibroblast to myofibroblast differentiation and to offer a therapeutic option for the treatment of fibrotic diseases. In this study, we addressed the role of NOX4 in physiological wound healing and in TGF-β-induced myofibroblast differentiation. We explored the phenotypic changes induced by TGF-β in primary skin fibroblasts isolated from *Nox4*-deficient mice by immunofluorescence, Western blotting and RNA sequencing. Mice deficient for *Cyba*, the gene coding for p22^phox^, a key subunit of NOX4 were used for confirmatory experiments as well as human primary skin fibroblasts. *In vivo*, the wound healing was similar in wild-type and *Nox4*-deficient mice. *In vitro*, despite a strong upregulation following TGF-β treatment, *Nox4* did not influence skin myofibroblast differentiation although a putative NOX4 inhibitor GKT137831 and a flavoprotein inhibitor diphenylene iodonium mitigated this mechanism. Transcriptomic analysis revealed upregulation of the mitochondrial protein *Ucp2* and the stress-response protein *Hddc3* in *Nox4*-deficient fibroblasts, which had however no impact on fibroblast bioenergetics. Altogether, we provide extensive evidence that NOX4 is dispensable for wound healing and skin fibroblast to myofibroblast differentiation, and suggest that another H_2_O_2_-generating flavoprotein drives this mechanism.

## Highlights

• NOX4 expression is upregulated during fibroblast to myofibroblast differentiation.
• NOX4 has no impact on mouse skin wound healing *in vivo*.
• TGF-β-induced NOX4 upregulation does not drive skin myofibroblast differentiation.
• Nox4-deficient fibroblasts shows upregulation of redox regulators *Ucp2* and *Hddc3*.
• Nox4 has no impact on skin fibroblast bioenergetics.

## 1 Introduction

Skin wound healing is a complex process comprising several overlapping phases and involving an orchestrated engagement of distinct cytokines, growth factors, and cell types. After an insult leading to the rupture of a blood vessel, repair mechanisms are initiated within seconds in order to control the hemorrhage via hemostasis and formation of the blood clot. Hemostasis is followed by an inflammation phase, which evolves into a proliferation phase characterized by the formation of a granulation tissue generated by fibroblast-derived cells, known as myofibroblasts, releasing extracellular matrix (ECM). The granulation phase is also mediated by immune cells and characterized by formation of a new network of vessels (neoangiogenesis). Re-epithelization, contraction, and tissue remodeling terminate the wound healing to form a scar [1,2].

Fibroblasts and myofibroblasts are essential cells for the replacement of the damaged tissue and contraction of the wound [3]. Although epithelial cells, endothelial cells, and bone marrow-derived circulating precursors (fibrocytes) are able to differentiate into myofibroblasts [4,5], it is well established that a local fibroblast population differentiates into myofibroblasts during the healing process and situations of tissue remodeling including fibrotic diseases, such as pulmonary, renal and liver fibrosis [6,7]. Fibroblasts and myofibroblasts produce growth factors and cytokines favoring migration, proliferation, and differentiation of several cell types involved in wound healing [8].

Insufficient differentiation of fibroblasts leads to prolonged open wounds (chronic wounds) while exaggerated myofibroblasts activity promotes hypertrophic scar formation and fibrosis [9]. Abnormal myofibroblast activity is not restricted to the skin, but also plays a central role in pathogenic mechanisms during fibrosis of various organs, such as lungs, heart, liver, and kidneys [10]. Moreover, a high density of myofibroblasts in the tumor environment correlates with poor prognosis in solid tumors [11].

Myofibroblast differentiation consists of a phenotypic change of fibroblasts, characterized by an increased expression of alpha-smooth muscle actin (α-SMA), contraction capabilities as well as enhanced production of ECM proteins, such as collagens, fibronectin, and elastin. Upon activation, fibroblasts first transform into middle-stage proto-myofibroblasts and then differentiate into mature myofibroblasts. Resting fibroblasts are spindle-shaped, while mature myofibroblasts are large cells with ruffled membranes [6,7,[12], [13], [14]].

Activation of myofibroblasts is regulated by numerous factors. A variety of cytokines, chemokines, growth factors, as well as reactive oxygen species (ROS), participate in myofibroblast differentiation [15]. TGF-β is a key cytokine controlling fibroblasts activation [16]. The TGF-β superfamily comprises three isoforms: TGF-β1 and TGF-β2 promote the conversion of fibroblasts into myofibroblasts, while TGF-β3 inhibits myofibroblast differentiation [17]. TGF-β1 treatment of human lung fibroblasts leads to hydrogen peroxide (H_2_O_2_) production [18] and upregulation of the H_2_O_2_-generating enzyme NADPH oxidase isoform 4 (NOX4) [19,20]. NOX are membrane-associated enzymes, which catalyze the formation of the superoxide radical anion (O_2_^•-^) and H_2_O_2_. Seven isoforms (NOX1-5, DUOX1-2) are found in humans [21]. NOX4 attracted much attention for its role in the transition of fibroblast to myofibroblasts and its profibrotic role [[19], [20], [21], [22], [23], [24]]. NOX4 has the highest expression in kidney proximal tubular cells, but moderate levels of NOX4 can be found in endothelial cells, epithelial cells, and myofibroblasts [25]. Upon expression, NOX4 constitutively generates H_2_O_2_ [26,27]. NOX4 activity requires the small membrane protein p22^phox^ for maturation and stabilization of a functional complex at the membrane [28]. While the role of NOX4 is extensively described in pathological states, such as fibrotic diseases [29], cancer [30], and cardiovascular disease [31], its physiological role is not known.

In this study, we aimed at exploring the role of NOX4 in skin wound healing and more specifically during fibroblast differentiation into myofibroblasts as follow-up of a previous study showing that Nox4 promoted wound healing in the mouse [32]. For this, we repeated an *in vivo* study to investigate the physiological wound healing process in *Nox4*-deficient mice. However, in this new study, we did not confirm a role for NOX4 in wound healing. Thus, we carried out an extensive *in vitro* characterization using primary skin fibroblasts from *Nox4*-and *Cyba*-deficient mice, as well as human skin fibroblasts. Altogether, we found that NOX4 is strongly upregulated following stimulation by isoforms 1 and 2 of TGF-β, but was dispensable for fibroblast to myofibroblast differentiation. Transcriptomics analysis revealed that mitochondrial uncoupling protein 2 (Ucp2) and the HD domain-containing protein 3 (Hddc3), two proteins involved in cellular redox signaling [33,34], as well as immunoglobulin superfamily containing leucine rich repeat (Islr) were dysregulated in *Nox4*-deficient mice suggesting that NOX4 is involved in the redox fine-tuning of fibroblasts and a potential role in fibroblast cellular metabolism. However, we did not find a role of Nox4 in mitochondrial-derived respiration or glycolytic function of myofibroblasts.

## 2 Materials and methods

### 2.1 Animal skin wound model

Wild type (WT, C57BL/6; Animal Facility at University of Geneva) and *Nox4* knockout (Nox4 KO; B6.129-*Nox4*^*tm1Kkr*^/J, Jackson Laboratory, Stock No: 022996 [23]) 8-weeks old female mice were used for *in vivo* experiments. Animal experiments were approved by local veterinary authority – Direction Générale de la santé de Genève (Authorization number GE/9/19). WT and Nox4 KO mice were kept in conventional conditions. To establish WT and Nox4 KO mice, heterozygous mice were bred together. Then, WT and Nox4 KO littermates were bred to obtain separate litters of WT and Nox4 KO.

Complete anesthesia was performed with inhaled isoflurane (Piramal Healthcare) and the dorsal skin was shaved and disinfected with Lifo-Scrub® (B. Braun Melsungen AG). The delineation of the wound was drawn with the use of a round template of 1.5 cm diameter on the back of each mouse. The wound was created by the resection of the marked area and removing a skin piece containing dermis and epidermis. Next, the wound was covered with dressing film (3 M TEGADERM) to prevent drying and infection. After surgery, mice were transferred to separate cages and monitored until full recovery from anesthesia. Mice were kept separately until the end of the experiment. Wounds were inspected every day and the dressing film was changed every two days.

### 2.2 Measurement of wound repair

The wound repair process was evaluated by several parameters: (i) the time required to complete wound closure (WC), (ii) the size of the wound during the healing process, and (iii) the quantification of the epithelization and contraction process at the time of WC. The wounds were considered closed when full re-epithelization occurred. The size of the wounds was documented using digital photography at designed time points – including the day of the wound creation (day 0), days 3, 5, 7, 10 after wounding, and day of WC – and quantified using Fiji software [35]. The hairless skin observed at the wound area after closure was considered as the area healed by epithelization, while the area healed by contraction was calculated by subtracting the area healed by epithelization from the initial area of the created wound.

### 2.3 Histology and immunohistochemistry (IHC) staining

Mice were sacrificed by an intraperitoneal injection of pentobarbital (150 mg/kg) at designed time points – day 3, 5, 7, 10, and the day of WC (n = 3 per time point for WT and Nox4 KO mice). If necessary, re-grown hair was shaved before skin collection. Harvested tissue included the wound and the area surrounding the wound edges. Following collection, the skin samples were fixed using 4% formaldehyde for 24 h, rinsed twice in phosphate buffered saline (PBS), and kept in PBS at 4 °C. After fixation, tissues were dehydrated, embedded in paraffin, and cut to obtain 5 μm tissue sections. For immunostaining, sections were incubated overnight at 56 °C, deparaffinized and re-hydrated.

IHC was performed for α-SMA as previously described [36]. The antibody was diluted (1:400) in Dako Real Antibody Diluent (Dako). Slides were incubated with peroxidase blocking solution (Dako) for 5 min at room temperature (RT), washed 2 times with 1x Washing Buffer (Dako) for 5 min at RT, and incubated in a humidity chamber with primary antibody for 30 min at 37 °C. Slides were washed 2 times with 1x Washing Buffer for 5 min at RT and incubated with Dako EnVision + System HRP labeled anti-mouse polyclonal secondary antibody (Dako EnVision+, HRP, K4000) for 30 min at RT. After 2 washes with 1x Washing Buffer, the color was developed using AEC Substrate Kit (BioGenex). When reddish color has appeared, the reaction was stopped by washing the slides with 1x Washing Buffer. After IHC staining, sections were incubated with hematoxylin for 5 min at RT to obtain labeling of the nuclei. Finally, slides were washed vigorously with tap water and mounted with mounting water-based solution Aquatex (Milipore).

Standard hematoxylin-eosin (HE) and Masson's trichrome protocols were used to visualize cellular infiltration and collagen content in skin wounded tissue.

All stained slides were scanned using slide scanner Zeiss Axioscan.Z1. Scanned images were quantified using QuPath [37] and a script developed by the Bioimaging Core Facility (University of Geneva). Briefly, the region of interest was selected visually – in the case of HE and Masson's trichrome staining, full areas of the wound were selected, while for α-SMA staining only the area near the edge of the wound below the epithelial tongue was selected as this area is enriched with myofibroblasts. Then the colored area –– (purple color for HE, blue color for Masson's trichrome, and red color for α-SMA) –was selected and the area of stained pixels was quantified.

### 2.4 Primary fibroblasts

Primary fibroblasts from different origins were used: (i) primary mouse skin fibroblasts, isolated from WT and Nox4 KO mice as well as *Cyba* KO mice (p22^phox^ KO, A.B6 Tyr + -Cyba_nmf333_/J, Jackson Laboratory strain 005445); (ii) primary human skin fibroblasts, isolated from a healthy donor (hDFs) and a patient carrying a mutation in *CYBA* gene (p22 mut hDFs) [38]; and (iii) human foreskin fibroblasts CCD-1112Sk (HFF, ATCCCRL 2429).

### 2.5 Primary mouse skin fibroblasts isolation

Primary mouse skin fibroblasts were obtained using a two-step enzymatic digestion. Following sacrifice, the abdominal hair was shaved using an electric shaver and/or scalpel and the skin was disinfected with 70% ethanol. Isolation of the skin started with a small incision at the bottom of the abdomen. Isolated abdominal skin was manually separated from the fat tissue, washed roughly in PBS (GIBCO) supplemented with penicillin (1 U/mL; GIBCO) and streptomycin (1 mg/mL; GIBCO) (P/S), and transferred into a Petri dish with fresh PBS with P/S. The tissue was cut into small, square pieces with a scalpel and transferred into a tube containing Dulbecco's Modified Eagle Medium (DMEM; GIBCO) with P/S, and Liberase™ DH Research Grad solution (0.0325 Wunsch units/mL; Roche Diagnostics GmbH, Roche Applied Science) and incubated for 150 min at 37 °C. Following digestion, the pieces of skin tissue were transferred to a new tube containing trypsin (2.5 mg/mL) and ethylenediaminetetraacetic acid (EDTA, GIBCO) and digested for an additional 30 min in a water bath at 37 °C, with shaking every 5 min. Undigested tissue was discarded, and the supernatant containing fibroblasts was filtered (40 μm) and centrifuged at 1500 rpm for 5 min RT. After centrifugation, the supernatant was discarded and the cell pellet was resuspended in 2 mL DMEM, 10% fetal bovine serum (FBS; GIBCO), and P/S. The cell suspension was plated into a T25 flask (25 cm^2^, Falcon). After isolation, primary skin mouse fibroblasts cultures were expanded up to passage 3. At passage 3, cells were resuspended in 90% FBS and 10% DMSO, transferred in cryotubes in CoolCell® cell freezing container (BioCision) and frozen at −80 °C. Cryotubes were transferred to liquid nitrogen for long-term storage.

### 2.6 Maintenance of cells

Cultured fibroblasts were maintained in complete DMEM supplemented with 10% FBS and P/S in standard conditions: 37 °C, 5% CO_2_, 21% O_2_. Additionally, primary hDFs and p22 mut hDFs media were supplemented with 10 ng/mL epithelial growth factor (EGF, Bio-Connect) and 10 ng/mL FGF_2_ (ProSpec-Tany TechnoGene Ltd.). Cells were passaged every 5–7 days using trypsin (5 mg/mL)/EDTA (GIBCO) and used between passage 4 and 7.

### 2.7 Genotyping

The genotype of mice and primary skin mouse fibroblasts cultures was confirmed using polymerase chain reaction (PCR). DNA was isolated from ear skin biopsies or primary cells using DirectPCR Lysis Reagent (Viagen Biotech) containing 200 μg/mL proteinase K (Sigma). Both WT and KO sequences of the *Nox4* and *Cyba* genes were amplified with GoTaq® G2 Green Master Mix (Promega) and specific primers (Microsynth AG), (Supplementary Table 1). The products of the PCR reaction were separated on 1% agarose gel and visualized with UV light. The generated amplicons allowed distinguishing WT from KO: *Nox4* WT sequence generated a 196 bp band, *Nox4* KO, a 363 bp band, *Cyba* WT, a 240 bp band, and *Cyba* KO, a 395 bp band.

### 2.8 Fibroblast proliferation and viability

The proliferation rate of primary fibroblasts was investigated by counting cells upon 5-ethynyl-2′-deoxyuridine (EdU) labeling.

For manual counting, cells were detached from the plate using trypsin (5 mg/mL)/EDTA, incubated with 0.02% Trypan Blue solution (GIBCO) to label dead cells, and counted with a Neubauer chamber (Marienfeld). Cell viability was represented as the ratio of live cells to the total number of cells. For automated cell count, cells were detached from the plate using trypsin (5 mg/mL)/EDTA, transferred into Tali™ Cellular Analysis Slide (Life Technologies Corporation), and counted with LifeTech Tali™ Image-based Cytometer. For EdU labeling, cells were plated inside the micro drop formed by the surface tension of the complete medium on the coverslip. Following cell attachment to the coverslip, cells were starved overnight in a serum-free medium to synchronize the cell cycle. Following starvation, cells were cultured in a standard medium containing 10 μM EdU for 24h. EdU was incorporated into the DNA of actively proliferating cells. Cells were fixed, permeabilized, and incubated with EdU labeling solution (fluorescent azide). Nuclei were stained with 1 μg/mL 4′,6-diamidino-2-phenylindole (DAPI) to determine the total number of cells. The microscopy images were taken with Axiocam Fluo microscope and ZEN Lite software. Cells were counted based on macroscopy images with Fiji software. The proliferation rate was calculated as the ratio of EdU labeled cells to the total number of cells.

### 2.9 Myofibroblast differentiation

Primary mouse skin fibroblasts were differentiated for 24 h or 5 days using 10 ng/mL TGF-β2 in 4 mM HCl and 0.1% bovine serum albumin (BSA). Human primary fibroblasts were differentiated for 24 h using 2 ng/mL TGF-β1 (DMSO, 0.5%, BioLegend) together with either DMSO, 1 μM diphenylene iodonium (DPI, Sigma) or 40 μM GKT137831 (Cayman Chemical) as previously described [39].

### 2.10 Silencing of NOX4 expression with siRNA

NOX4 expression was silenced using small interfering RNA (siRNA) technology. NOX4 or nonspecific siRNA (siNOX4 1 – Thermofisher Scientific, ID: s224161, siCTRL – *Silencer*™ Select Negative Control No. 1 siRNA, Thermofisher Scientific #4390843) were used to transfect HFF. Cells were grown until 70% confluence HFF were transfected with 25 nM of control or NOX4 siRNA prepared in TransIT X2® Dynamic Delivery system (Mirus Bio) according to the manufacturer's protocol. TGF-β1 (2 ng/mL) was added immediately after transfection and cells were collected after 24 h for RNA extraction and qPCR.

### 2.11 Immunofluorescence (IF) microscopy

For α-SMA immunofluorescence staining, fibroblasts were fixed with 1% paraformaldehyde (PFA) for 30 min at RT, rinsed 3 times with PBS, and permeabilized with cold 100% methanol for 5 min at −20 °C. Fixed cells were washed 3 times with PBS and incubated for 1h, at RT with a primary antibody against α-SMA (1:200, [36]). Cells were washed 3 times with PBS and incubated with secondary anti-mouse IgG antibodies conjugated with either AlexaFluor 488 or AlexaFluor 568 (1:100, Jackson ImmunoResearch Laboratories, Inc) for 1h, at RT. Labeled cells were rinsed 3 times with PBS and mounted with ProTaqs MountFluor Anti-Fading containing DAPI (BIOCYC). The number of positively stained cells was counted on microscopy images using Fiji software and normalized to the total number of cells. For total actin visualization, filamentous (F)-actin was stained with phalloidin. Fibroblasts were fixed with 1% PFA for 30 min in RT, rinsed 3 times with PBS, and permeabilized with 0.01% Triton X-100 for 5 min in RT. Cells were washed 3 times with PBS and incubated with phalloidin conjugated with AlexaFluor 488 (1:200; Thermo Fisher Scientific Inc.) for 1h, at RT. Labeled cells were rinsed 3 times with PBS and mounted with ProTaqs MountFluor Anti-Fading containing DAPI (BIOCYC). The intensity of fluorescence was measured on microscopy images using Fiji software and normalized to the total number of cells. The IF microscopy was performed using Axiocam Fluo microscope and ZEN Lite software.

### 2.12 Contraction capabilities of myofibroblasts

Commercialized silicon dishes with the defined softness of 5 kPa (ExCellness Biotech SA) were used to measure the contraction capabilities of the primary skin fibroblasts. Cells were plated into standard plastic dishes and differentiated into myofibroblasts as described above. After the designed time, cells were transferred into a silicon dish and allowed to attach and contract for 6h. Cells were fixed with 1% PFA, rinsed 3 times with PBS, and incubated with 1 μg/mL DAPI for 10 min in RT to stain nuclei. Between 1000 and 2000 cells were analyzed for each experiment (n = 3). The total number of cells analyzed per condition was as follows: WT + vehicle 4621 cells; WT + TGF-β2: 3821 cells; Nox4 KO + vehicle: 5184 cells; Nox4 KO + TGF-β2: 3920. Contracted cells were recorded using phase-contrast microscopy at Video time-lapse microscope and ZEN Lite software. The number of wrinkling cells was normalized to the total number of cells and quantified using Fiji software.

### 2.13 H_2_O_2_ detection

H_2_O_2_ levels were measured using ROS-Glo™ H_2_O_2_ Assay (Promega Corporation) according to manufacturer instruction. Briefly, fibroblasts were plated on 96-well white plate with a clear bottom and cultured with or without 10 ng/mL TGF-β2 for 5 days. As control of the assay, tetracycline-inducible NOX4 overexpression system NOX4 T-Rex™ cells were incubated with tetracycline (1 μg/mL, Sigma) for 24h to induce NOX4 expression [40]. Detection of H_2_O_2_ was performed by incubating cells with H_2_O_2_ Substrate Solution for 6h at 37 °C. Then, cells were lysed with Detection Solution for 20 min in RT, after which luminescence was measured using SpectraMax L 96w at READS unit, University of Geneva, Switzerland.

### 2.14 Real-time quantitative polymerase chain reaction (qPCR)

RNA was extracted using a RNeasy mini kit (Qiagen) and quantified using a NanoDrop™ 2000 spectrophotometer (Thermo Scientific™). 500 ng of RNA was used for cDNA synthesis using SensiFAST cDNA Synthesis Kit (Bioline) following the manufacturer's instruction. Real-time PCR was performed using SYBR green assay on a 7900HT SDS system from ABI at the Genomics Platform, National Center of Competence in Research Frontiers in Genetics, Geneva. The efficiency of each primer was verified with serial dilutions of cDNA. Sequences of the primers are reported in Supplementary Table 2. Relative expression levels were calculated by normalization to the geometric mean of the two house-keeping genes, either EEF1 and GAPDH, or GADPH and RPLP0, as described previously [41].

### 2.15 Western blotting (WB)

Total protein extracts were obtained from fibroblasts using radioimmunoprecipitation assay (RIPA) buffer containing proteases and phosphatases inhibitors cocktail (Thermo Scientific). Briefly, primary skin fibroblasts were washed and lysed with RIPA buffer for 20 min on ice and cells were detached using a cell scraper. The cellular suspension was transferred into Eppendorf tubes and centrifuged for 20 min 14 000 rpm at 4 °C. The protein soluble fraction was transferred into new Eppendorf tube and stored at −20 °C. The concentration of isolated proteins was measured with Pierce™ BCA Protein Assay Kit (Thermo Scientific™). Equal amounts of proteins were separated using SDS-PAGE electrophoresis and transferred onto nitrocellulose membranes, which were blocked for 1h in RT with 5% powder milk or with 2% BSA (Sigma) dissolved in 0.01% Tween 20 in Tris-buffered saline (TBS-T). Membranes were incubated overnight at 4 °C with primary antibodies against α-SMA (1:500, [36]) or GAPDH conjugated with HRP (1:1000, Abcam) in 5% milk TBS-T. Secondary anti-mouse IgG antibody was incubated in 5% milk TBS-T (1:3000, Bio-Rad Laboratories Inc.) for 1h in RT. For detection, membranes were incubated with SuperSignal™ West Pico PLUS Chemiluminescent Substrate (Thermofisher) and the images were taken with Fusion software (Witec AG).

### 2.16 RNA sequencing (RNAseq)

cDNA libraries were constructed from 300 ng of total RNA isolated as described before by the Genomic platform of the University of Geneva using the Illumina TruSeq RNA Sample Preparation Kit according to the manufacturer's protocol. Libraries were sequenced using single-end (50 nt-long) on Illumina HiSeq4000. FastQ reads were mapped to the ENSEMBL reference genome (GRCm38.96) using STAR version 2.4.0j [42] with standard settings, except that any reads mapping to more than one location in the genome (ambiguous reads) were discarded (m = 1). A unique gene model was used to quantify reads per gene. Briefly, the model considers all annotated exons of all annotated protein coding isoforms of a gene to create a unique gene where the genomic region of all exons is considered coming from the same RNA molecule and merged together. All reads overlapping the exons of each unique gene model were reported using feature Counts version 1.4.6- p1 [43]. Gene expressions were reported as raw counts and in parallel normalized in RPKM in order to filter out genes with low expression value (1 RPKM) before calling for differentially expressed genes. Library size normalizations and differential gene expression calculations were performed using the package edgeR [44] designed for the R software [45]. Only genes having a significant fold-change (Benjamini-Hochberg corrected p-value <0.05) were considered for the RNA seq analysis. Data containing significantly upregulated and downregulated genes from RNA seq analysis were uploaded to the STRING application (https://string-db.org/) [[46], [47], [48], [49], [50], [51], [52], [53], [54], [55], [56]]. The pathway analysis was performed to identify regulated pathways that were associated with significantly changed mRNA expression obtained in RNA seq. The section, with identified pathways involved in regulation of biological processes, was extracted, and the most 10 relevant pathways involved in myofibroblast differentiation were included in the graph.

### 2.17 Determination of the ratio of mitochondrial DNA to genomic DNA

Total DNA was isolated using DNeasy Blood & Tissue Kits (QIAGEN) according to manufacturer protocol. DNA concentration was measured with NanoDrop™ 2000 spectrophotometer (Thermo Scientific™). Four ng of DNA was used for qPCR. The ratio of mitochondrial DNA to genomic DNA was determined as CT values of mitochondrial genes (Mt-Nd1, Mt-Cox1, Mt-Atp6) divided by Ct value for Gadph as described [57].

### 2.18 Real time cell metabolic analysis (Seahorse XFe96 analyzer)

Cell metabolism was analyzed using two assays – Seahorse XF Cell Mito Stress Test (Agilent) and Seahorse XF Glycolysis Stress Test (Agilent). In Seahorse XF Cell Mito Stress Test, oxidative phosphorylation was investigated, while in Seahorse XF Glycolysis Stress Test, glycolysis capacity after glucose deprivation was examined.

In both cases, 30 000 cells were plated into Seahorse XF Cell Culture Plates. Cells were starved (no FBS) for 24h and cells were treated with 10 ng/mL TGF-β2 for another 24 h in complete medium. Before each assay, XFe96 cartridge was hydrated overnight in water in a no-CO_2_ incubator at 37 °C. On the day of measurement, water was replaced by pre-heated Seahorse XF Calibrant for 90 min. Cell medium was removed and replaced by 180μl/well Agilent Seahorse XF DMEM Medium pH 7.4 (# 103575–100) and kept for 1 h in a no-CO_2_ incubator at 37 °C.

In Seahorse XF Cell Mito Stress Test, three different compounds were added sequentially to modulate oxidative phosphorylation. Firstly, oligomycin (3 μM final) was added to block ATP-synthase, which is characterized by the decreased in electron transfer through the mitochondrial electron transport chain and reduce oxygen consumption by mitochondria. Secondly, carbonyl cyanide-4 (trifluoromethoxy) phenylhydrazone (FCCP) (1 μM final) was injected. FCCP is an uncoupling compound, which destroys the electron transport chain and leads to maximal used of oxygen by complex IV, which is considered as maximal mitochondrial respiration. In the last step, a mix of rotenone and antimycin A (0.5 μM final) was added, which leads to complete blocking of mitochondrial complexes III and I, and results in the irreversible termination of mitochondrial respiration. Oxygen consumption rate (OCR) and extracellular acidification rate (ECAR) were measured during the assay.

In the first step of the Seahorse XF Glycolysis Stress Test, cells were deprived of glucose for 1h. Then three different compounds were added respectively. Firstly, glucose (10 mM final) was added to begin the glycolysis in the cells. Then, oligomycin (3 μM) was injected to block ATP-synthase, considered as inhibitor of mitochondrial respiration to force cells to shift cellular metabolism more into glycolysis. In the last step, 2-deoxy-glucose (2-DG; glucose analog) (25 mM final) was injected to bind to hexokinase and block glucose metabolism. As in the Seahorse XF Cell Mito Stress Test, both OCR and ECAR were measured.

At the end of each experiments, cells stained with Hoechst (20 μg/mL) for 30 min at 37 °C. Total cell number was counted automatically with Cytation 5 Cell Imaging Multi-Mode Reader (Agilent) at READS unit, University of Geneva, Switzerland.

## 3 Results

### 3.1 Deletion of NOX4 has no impact on physiological skin wound healing

**Fig. 1:**
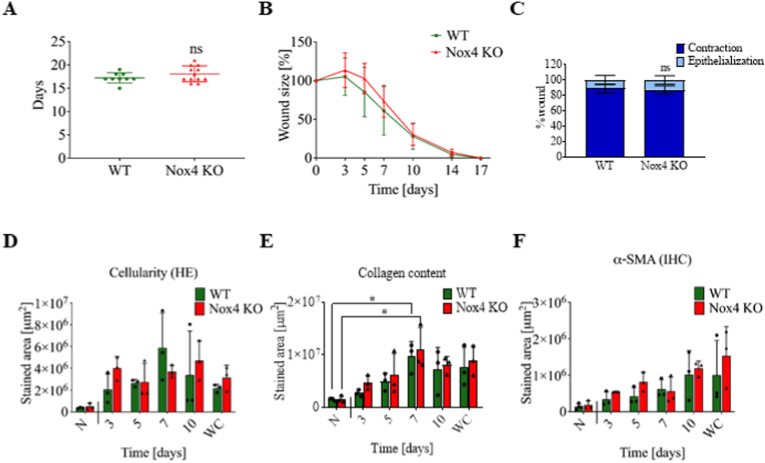
Physiological skin wound repair was not affected by *Nox4* deficiency *in vivo*. A. Time for complete wound closure in WT and Nox4 KO mice, each dot corresponds to one mouse; B. Wound size as a function of time normalized to initial size of the wound; C. Epithelization/contraction ratio; D. Quantification of hematoxylin stained area indicating cellular infiltration in the wounds over time until complete wound closure (WC); E. Quantification of Masson's trichrome staining as a measure of collagen content in the wounds over time until complete WC; F. α-SMA-positive area in the wounds over time until complete WC; N – normal, unwounded skin, WC – wound closure; *in vivo* experiments *–* WT n = 9, Nox4 KO n = 12; IHC staining WT n = 3, Nox4 KO n = 3 for each time point. Data shown as mean ± SD; ns – not significant; *p < 0.05; Two-way ANOVA.

**Supplementary Fig. 1:**
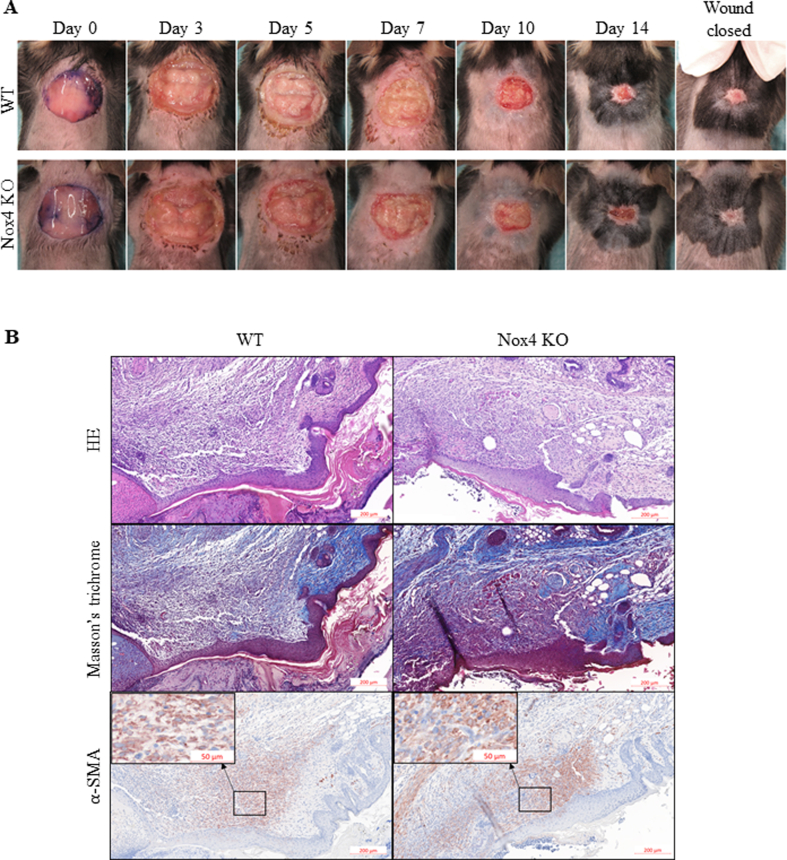
Physiological wound repair in WT and Nox4 KO mice. A. Representative digital images of skin wounds at day 0, 3, 5, 7, 10, 14 and wound closure from WT and Nox4 KO mice; B. Representative images of HE staining, Masson’s trichrome staining and IHC staining for α-SMA at day 7 of wound healing in WT and Nox4 KO mice.

To investigate the role of Nox4 in physiological skin wound healing, we used an *in vivo* mouse model of wound repair. Experimentally, we generated rounded wounds (1.5 cm diameter) at the back of 8-weeks old female WT and Nox4 KO mice. The wounds took approximately 15 days for complete wound closure for both WT and Nox4 KO mice (Fig. 1A). Wound closure started after 3 days and followed a similar pattern in WT and Nox4 KO mice (Fig. 1B, Supplementary Fig. 1A). Contraction by myofibroblasts and *de novo* synthesis of epithelium were also quantified indicating a predominant role of myofibroblast contraction and a minimal impact of re-epithelization, but no difference between WT and Nox4 KO for both parameters (Fig. 1C). The primary response after wounding involves the infiltration of inflammatory cells followed by fibroblast activation and myofibroblast differentiation. HE staining showed that cellular infiltration increased rapidly after the wound induction and stabilized at day 7 (Fig. 1D, Supplementary Fig. 1B HE). Masson's trichrome staining, which labels collagen (blue) and keratin (red), indicated that collagen content significantly peaked at day 7 (Fig. 1E, Supplementary Fig. 1B Masson's trichrome). Detection of α-SMA showed a constant increase of number of myofibroblasts until wound closure (Fig. 1F, Supplementary Fig. 1B α-SMA). This pattern is consistent with a typical course of wound healing process but showed no difference between WT and Nox4 KO mice.

### 3.2 Nox4 is upregulated in mouse skin fibroblasts following TGF-β1 and 2 treatment, but does not contribute to myofibroblast differentiation *in vitro*

**Fig. 2:**
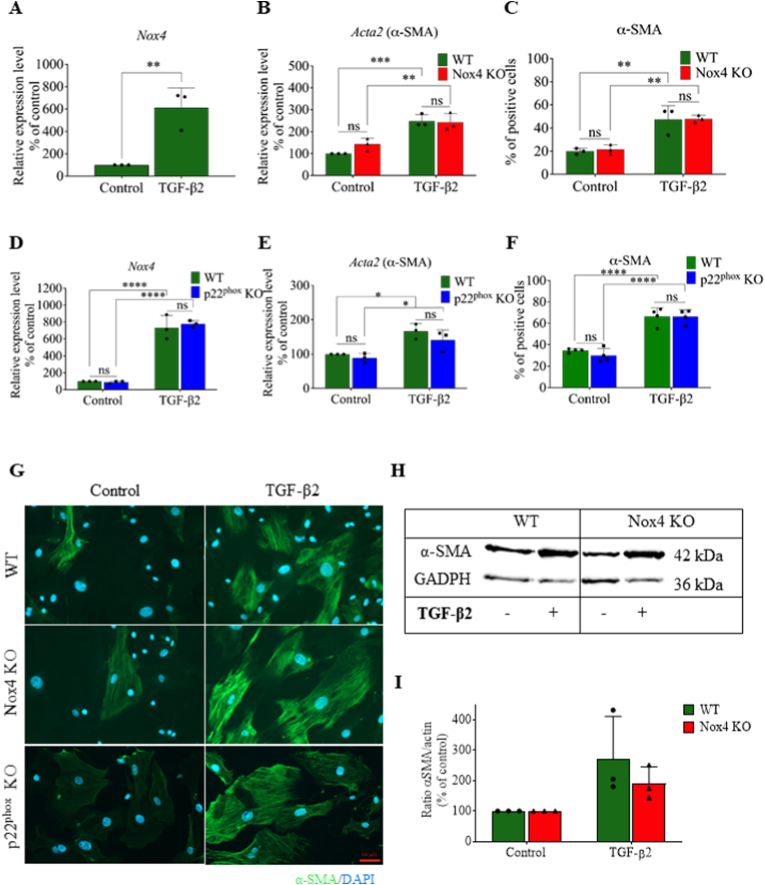
*Nox4* deficient primary mouse skin fibroblasts were able to differentiate into myofibroblasts after TGF-β2 stimulation. A. *Nox4* mRNA expression in WT primary skin fibroblasts cultured without (Control) or with TGF-β2; B. *Acta2* mRNA expression in WT and Nox4 KO primary skin fibroblasts cultured without or with TGF-β2; C. Percentage of α-SMA-positive cells in WT and Nox4 KO primary skin fibroblasts cultured without or with TGF-β2; D. *Nox4* mRNA expression in WT and p22^phox^ KO primary skin fibroblasts cultured without or with TGF-β2; E. *Acta2* mRNA expression in WT and p22^phox^ KO primary skin fibroblasts cultured without or with TGF-β2; F. Percentage of α-SMA-positive cells of WT and p22^phox^ KO primary skin fibroblasts cultured without or with TGF-β2; G. Representative immunofluorescence staining of α-SMA (green) and DAPI (blue) in WT, Nox4 KO and p22^phox^ KO primary skin fibroblasts cultured without or with TGF-β2; H. Representative Western blot for α-SMA in WT, Nox4 KO; I. Quantification of α-SMA band intensity from primary skin fibroblasts cultured without or with TGF-β2 in WT and Nox4 KO primary skin fibroblasts, GADPH was used as loading control; WT (green bars) n = 3, Nox4 KO (red bars) n = 3; Data shown as mean ± SD; ns – not significant, *p < 0.05, **p < 0.01, ***p < 0.001, ****p < 0.0001; Two-way ANOVA. (For interpretation of the references to color in this figure legend, the reader is referred to the Web version of this article.)

**Supplementary Fig. 2:**
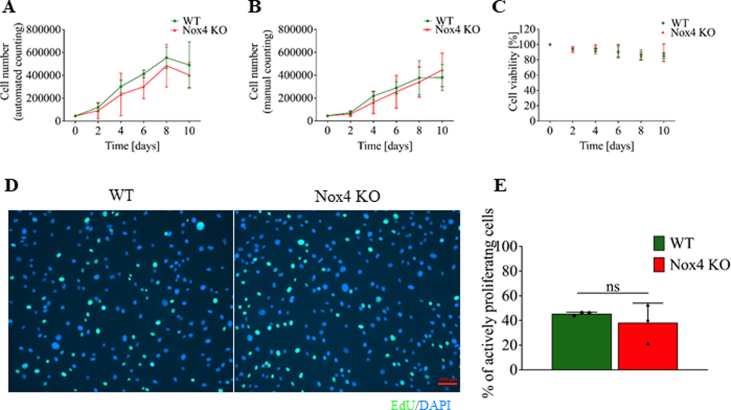
Nox4 deficiency did not affect basal survival and proliferation capacity of primary skin fibroblasts. A. Number of WT and Nox4 KO primary skin fibroblasts counted automatically every 2 days during 10 days of standard cell culture; B. Number of WT and Nox4 KO primary skin fibroblasts counted manually every 2 days during 10 days of standard cell culture; C. Viability of WT and Nox4 KO primary skin fibroblasts represented as a percentage of Trypan blue negative cells to the total number of cells; D. Representative fluorescent images of WT and Nox4 KO primary skin fibroblasts labelled with EdU (green) and stained with DAPI (blue); E. Proliferation rate of WT and Nox4 KO primary skin fibroblasts represented as percentage of actively proliferating cells (EdU positive) to the total number of cells; WT (green circles, lines and bars) n = 3, Nox4 KO (red triangles, lines and bars) n = 3; Data shown as mean ± SD; ns – not significant; Cell number, viability – Two-way ANOVA, Proliferation capacity – Unpaired t-test.

**Supplementary Fig. 3:**
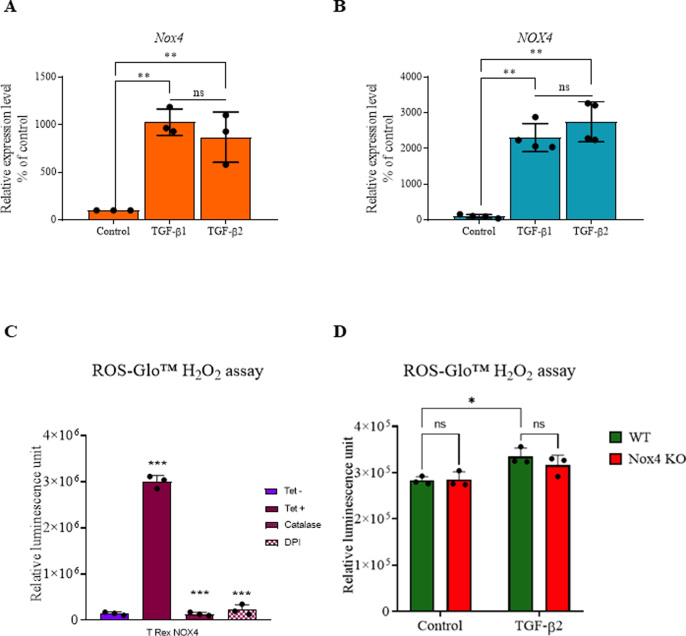
TGF-β treatment led to increased NOX4 mRNA expression and ROS generation in human skin fibroblasts. A. and B. NOX4 mRNA expression is upregulated in primary mouse and human skin fibroblasts cultured without (Control) or with TGF-β1 or TGF-β2. C. and D. ROSGloTM H2O2 assay. Expression of NOX4 was induced by tetracycline (Tet) leading to increased luminescence. The signal is inhibited by catalase and DPI. D. Detection of H2O2 in skin fibroblasts differentiated by TGF-β2 (5 days) showed low luminescence levels compared to Tet-induced NOX4 cells (C.) and no difference between WT and Nox4. Data shown as mean ± SD; *** p < 0.001, ### p < 0.0001; Two-way ANOVA.

To test the potential impact of Nox4 on myofibroblast differentiation, we measured the expression of α-SMA in TGF-β2-treated primary mouse skin fibroblasts using two distinct knockout mice, namely Nox4 KO and p22^phox^ KO mice. The p22^phox^-deficient mice were chosen because the p22^phox^ protein is essential for the function of Nox4 as well as Nox1, Nox2, and Nox3 [58,59]. We first showed that TGF-β1 and TGF-β2 were equivalent in inducing Nox4 expression (approximately 10 times) after 24h in primary mouse skin fibroblasts (Supplementary Fig. 3A), suggesting that the upregulation of Nox4 is mediated by a similar mechanism of action following TGF-β1 and TGF-β2. Moreover, Nox4 expression remained upregulated after 5 days of TGF- β2 stimulation (Fig. 2A and D). NOX4 upregulation was previously described following TGF-β1 in several other types of fibroblasts [19,20,60]. We measured a similar increase of α-SMA in WT, Nox4 KO, and p22^phox^ KO primary fibroblasts after TGF-β2 stimulation by qPCR (*Acta2* gene; Fig. 2B and E) protein levels by IF (Fig. 2C, F, and 2G), thereby confirming our *in vivo* data. Similar TGF-β2-mediated increase of α-SMA in fibroblasts from WT and Nox4 KO mice was confirmed by WB (Fig. 2H and I). To exclude a potential intrinsic difference between WT and Nox4 KO fibroblasts, we verified that WT and Nox4 KO fibroblasts *in vitro* had similar cellular growth (Supplementary Figs. 2A and 2B), viability (Supplementary Fig. 2C), proliferation capacity (Supplementary Figs. 2D and 2E) and H_2_O_2_ generation (Supplementary Figs. 3C and D).

**Fig. 3:**
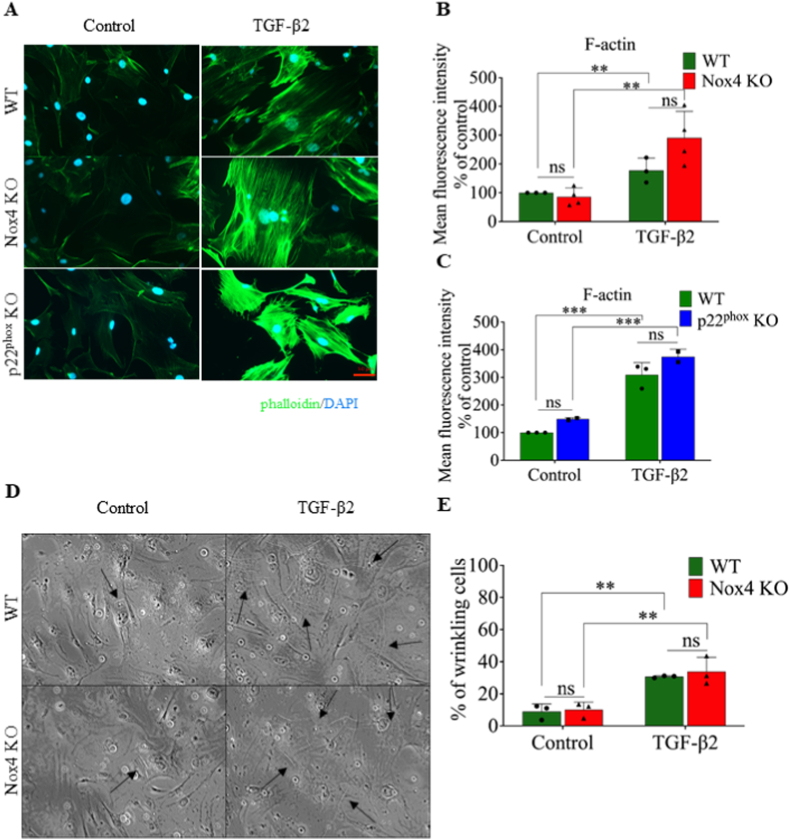
Nox4 did not influence morphology of cytoskeleton and contraction capabilities of primary mouse skin fibroblasts differentiated into myofibroblasts after TGF-β2 stimulation. A. Representative immunofluorescence staining for F-actin labeled with phalloidin (green) in WT, Nox4 KO and p22^phox^ KO primary skin fibroblasts cultured without or with TGF-β2; nuclei are stained with DAPI (blue); B and C. Quantification of the content of F-actin in WT, Nox4 KO (B) and p22^phox^ (C) primary skin fibroblasts cultured without or with TGF-β2; D. Representative images of wrinkles made on silicon of softness with E = 5 kPa by WT and Nox4 KO primary skin fibroblasts cultured without or with TGF-β2, arrows indicates wrinkles made by cells; E. Percentage of wrinkling WT and Nox4 KO primary skin fibroblasts cultured without or with TGF-β2; WT (green bars) n = 3, Nox4 KO (red bars) n = 3, p22^phox^ KO (blue bars) n = 3; Data shown as mean ± SD; ns – not significant, **p < 0.01, ***p < 0.001; Two-way ANOVA. (For interpretation of the references to color in this figure legend, the reader is referred to the Web version of this article.)

To examine the impact of Nox4 on the general cytoskeleton morphology, we performed fluorescently conjugated phalloidin staining to detect total F-actin. Phalloidin staining was significantly increased in WT, Nox4 KO, and p22^phox^ KO primary fibroblasts following TGF-β2 treatment (Fig. 3A, **3B**, **3C**) and displayed tight, oriented F-actin distribution typical of myofibroblasts (Fig. 3A), confirming that formation of F-actin was typical in myofibroblasts despite the absence of a functional Nox4 complex.

We investigated the contraction capabilities of fibroblasts. TGF-β2 induced cellular contraction of fibroblasts, but we did not observe a difference in contraction between WT and Nox4 KO primary fibroblasts, suggesting that the functionality of fibroblasts is preserved in spite of Nox4 deficiency (Fig. 3D, **E**).

Altogether, an in-depth *in vitro* analysis using mouse primary skin fibroblasts showed that genetic deficiency of two subunits necessary for Nox4 activity did not mitigate normal fibroblast-to-myofibroblast differentiation.

### 3.3 NOX4 is upregulated in human skin fibroblasts following TGF-β1 treatment, but does not contribute to myofibroblast differentiation *in vitro*

To verify that the above observations were not limited to genetically modified mice, we silenced NOX4 expression by siRNA in human primary skin fibroblasts stimulated with TGF-β1.

**Fig. 4:**
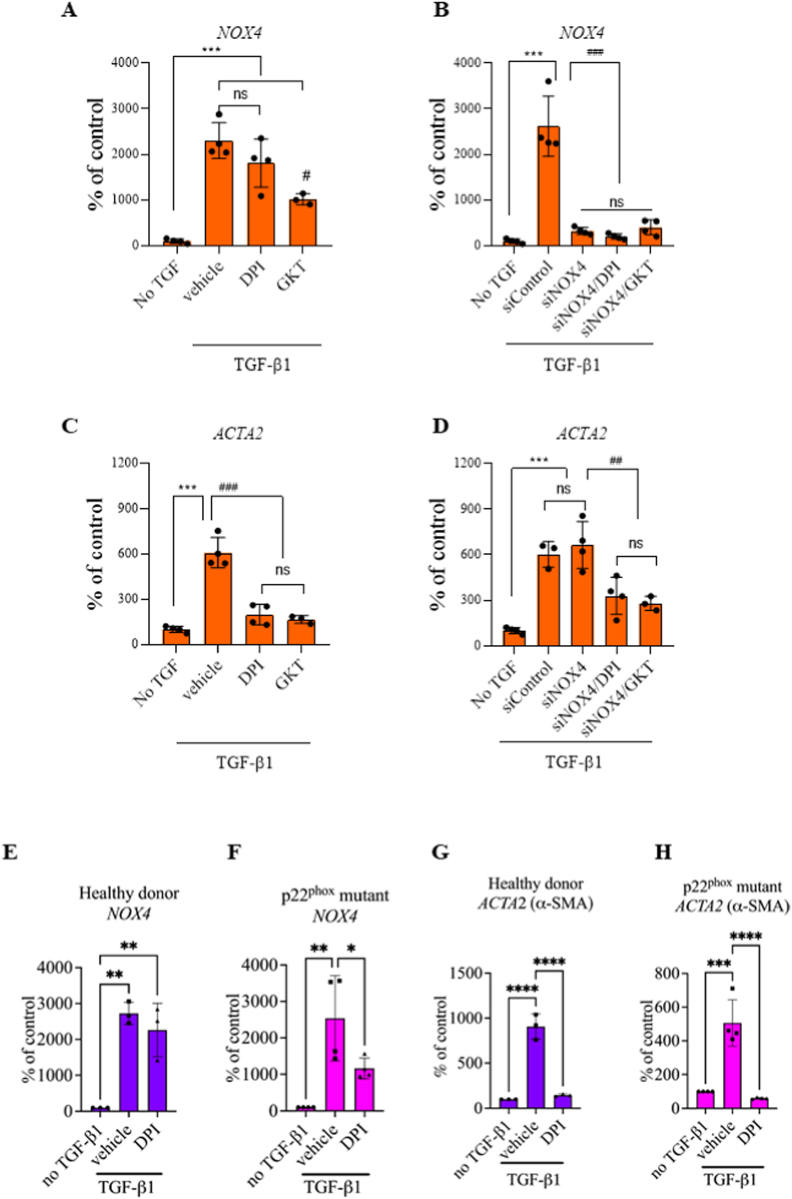
NOX4 did not drive myofibroblast differentiation of human primary fibroblasts. A and B. *NOX4* mRNA expression after treatment with DPI 1 μM and GKT137831 40 μM (A) and siRNA silencing of NOX4 gene ± compounds (B) in HFF cultured without or with TGF-β1; C and D. *ACTA2* mRNA expression after treatment with DPI 1 μM and GKT137831 40 μM (C) and siRNA silencing of NOX4 gene ± compounds (D) in HFF cultured without or with TGF-β1; E and F *NOX4* mRNA expression in hDFs (E) and p22^phox^ mutated hDFs (F) cultured without or with TGF-β1 and DPI; G. and H. *ACTA2* mRNA expression in hDFs (G) and p22^phox^ mutated hDFs (H) cultured without or with TGF-β1 and DPI; HFF, n = 3; hDFs, n = 3; p22^phox^ mutated hDFs, n = 3 or 4. Data shown as mean ± SD; ns – not significant, **p < 0.01, **p < 0.01, ***p < 0.001, ****p < 0.0001; One-way ANOVA.

Both TGF-β1 and TGF-β2 induced a significant and comparable (approximately 20 times) upregulation of NOX4 mRNA in human primary fibroblasts (Supplementary Fig. 3B). Treatment with GKT137831, a putative NOX1/NOX4 inhibitor slightly mitigated NOX4 upregulation, but the broad-spectrum NOX inhibitor DPI did not alter NOX4 upregulation (Fig. 4A). The siRNA directed against NOX4 significantly downregulated NOX4 expression nearly to the basal level (Fig. 4B). TGF-β1 significantly increased α-SMA expression in human skin fibroblasts, which was significantly mitigated by GKT137831 and DPI (Fig. 4C). However, transfection with either control siRNA or two different siRNAs against NOX4 did not change α-SMA expression. Interestingly, addition of GKT137831 or DPI significantly decreased α-SMA expression, suggesting an alternative mode of action of these compounds (Fig. 4D).

To confirm these observations, we compared primary human dermal fibroblasts isolated from a patient with a mutation in the *CYBA* gene (p22 mutated hDFs) and a healthy donor (hDFs). Primary fibroblasts were stimulated with TGF-β1 for 24 h and mRNA expression of NOX4 and ACTA2 (α-SMA gene) were measured. TGF-β1 significantly upregulated NOX4 expression in hDFs and p22 mutated hDFs (Fig. 4E and F). Similar to p22^phox^ KO mouse fibroblasts, both hDFs and p22 mutated hDFs showed similar upregulation of ACTA2 expression following TGF-β1 as shown by qPCR while DPI decreased ACTA2 expression in both p22 mutated hDFs and hDFs (Fig. 4G and H). Intriguingly, DPI mitigated NOX4 upregulation in p22 mutated hDFs, but not in healthy fibroblasts.

Altogether, the *in vitro* studies performed on the mouse and human primary skin fibroblasts confirmed that NOX4 activity does not drive myofibroblast differentiation and suggest that GKT137831 and DPI target alternative pathways responsible for myofibroblast differentiation.

### 3.4 NOX4 deficiency leads to upregulation of Ucp2 and Hddc3 in mouse fibroblasts

**Fig. 5:**
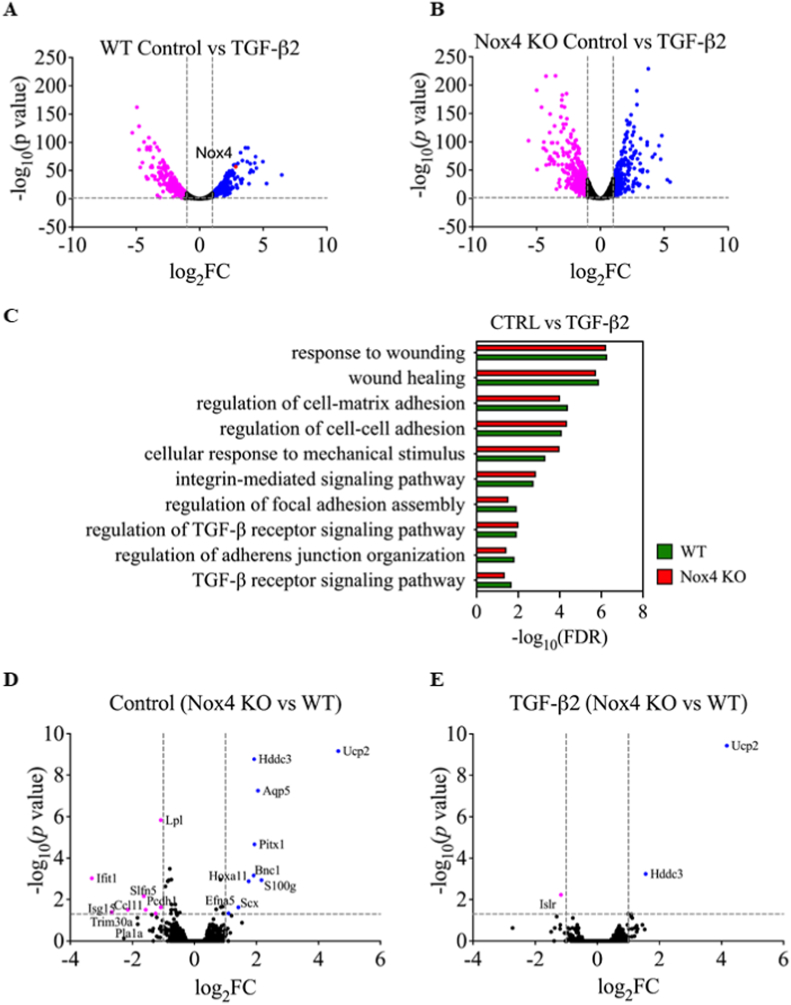
*Nox4* deficiency led to up-regulation of *Ucp2* and *Hddc3* and down-regulation of *Islr* in primary mouse skin fibroblasts. A and B. Volcano plots representing RNA sequencing data comparing of TGF-β2-stimulation to control conditions in WT (A) and Nox4 KO (B) primary skin fibroblasts; C. Graph representing different pathways involved in biological processes activated by TGF-β2 stimulation of WT and Nox4 KO fibroblasts; D and E. Volcano plots representing RNA sequencing data comparing Nox4 KO to WT primary skin fibroblasts in control conditions (D) and after TGF-β2 stimulation (E); significantly upregulated genes (blue dots), significantly downregulated genes (pink dots); WT – green bars; Nox4 KO – red bars; WT n = 3; Nox4 KO n = 3; Data shown as –log_10_ of false discovery rate; fold-change with Benjamini-Hochberg correction; p < 0.05. (For interpretation of the references to color in this figure legend, the reader is referred to the Web version of this article.)

To investigate the biological significance of the induction of Nox4 expression after TGF-β stimulation, we performed global RNAseq using WT and Nox4 KO mouse primary skin fibroblasts stimulated with TGF-β2 for 24h. We observed that TGF-β2 dramatically modified the transcriptome of both WT and Nox4 KO fibroblasts (Fig. 5A, **5B**). In WT fibroblasts stimulated with TGF-β2, 283 genes were significantly upregulated, including Nox4, and 575 were downregulated. In Nox4 KO fibroblasts, 260 genes were upregulated, and 569 genes were downregulated.

**Supplementary Fig. 4:**
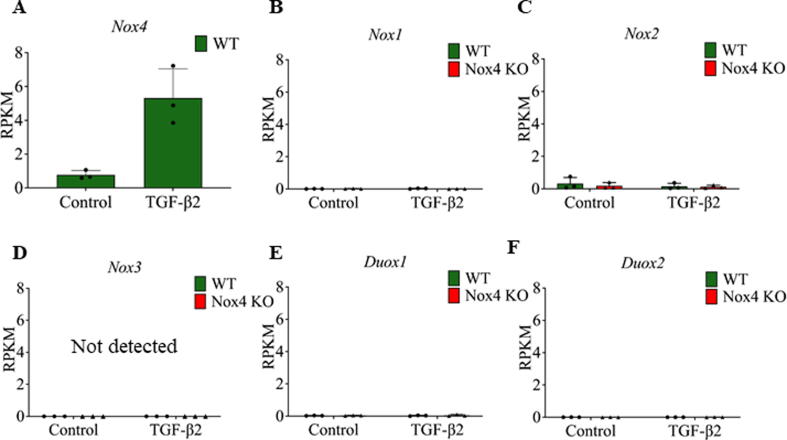
Expression of Nox isoforms in RNA seq dataset. A to F. mRNA expression level of different Nox isoforms – Nox4 (A), Nox1 (B), Nox2 (C), Nox3 (D), Duox1 (E), and Duox2 (F) in WT and Nox4 KO primary skin fibroblasts in control condition or after 24h of TGF-β2 stimulation determined by RNA sequencing; WT (green bars) n = 3, NOX4 KO (red bars) n = 3, RPKM – Reads per kilo base per million mapped reads; Data shown as mean ± SD.

**Supplementary Fig. 5:**
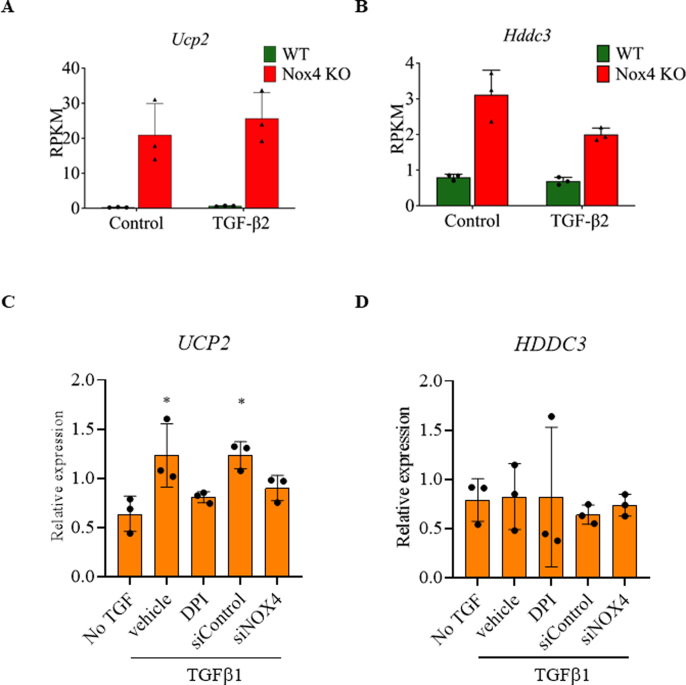
Differential expression of Ucp2 and Hddc3 in primary mouse and human skin fibroblasts. A. and B. mRNA expression of Ucp2 (A), Hddc3 (B) in WT and Nox4 KO primary skin fibroblasts in control condition or after 24h of TGF-β2 stimulation determined by RNA sequencing; WT (green bars) n = 3, Nox4 KO (red bars) n = 3, RPKM – Reads per kilo base per million mapped reads; Data shown as mean ± SD. C and D. UCP2 (C) and HDDC3 (D) mRNA expression after DPI treatment (1 μM) and siRNA silencing of NOX4 gene in HFF cultured without or with TGF-β1.

Pathways analysis confirmed the dysregulation of characteristic pathways involved in myofibroblast differentiation, such as wound healing, TGF-β signaling pathway, SMAD signaling, ECM and cytoskeleton regulation in both WT and Nox4 KO following TGF-β2 (Fig. 5C). Direct comparison of WT and Nox4 KO fibroblasts highlighted only very few differentially expressed genes, as 9 genes were significantly upregulated (≥2-fold) and 8 genes significantly downregulated (≥2-fold) out of a total of 10277 genes (Fig. 5D). Following TGF-β2 stimulation, only 2 genes were significantly upregulated and one gene was significantly downregulated out of a total of 9945 evaluated genes in NOX4 KO fibroblasts when compared to WT fibroblasts (Fig. 5E). The RNAseq analysis revealed that Nox4 KO fibroblasts showed a strong upregulation of the uncoupling protein 2 (*Ucp2*) gene (Supplementary Fig. 5A) and the HD domain containing 3 (*Hddc3)* gene (Supplementary Fig. 5B) even without TGF-β2 stimulation as well as downregulation immunoglobulin superfamily containing leucine rich repeat (Islr) gene after TGF-β2 stimulation (Fig. 5E). The complete list of significantly up- or downregulated genes in all conditions are documented in GEO database under the accession number GSE197562. Such minimal changes in transcriptome between WT and Nox4 primary fibroblasts were not expected. To exclude a compensatory mechanism of another NOX isoform in Nox4 KO fibroblasts, we extracted the expression levels of all 6 murine Nox isoforms in the RNAseq dataset. None of the Nox isoforms were upregulated in Nox4 KO fibroblasts, showing low expression levels in WT and Nox4 KO fibroblasts (Supplementary Figs. 4A–F).

In order to address a possible impact of NOX4 on *UCP2* and *HDDC3* expression in human cells, we used HFF stimulated with TGF-β1 treated with siRNA or DPI and performed qPCR. We found a different pattern of expression in humans compared to mouse skin fibroblasts. TGF-β1 upregulated UCP2, while DPI and NOX4 siRNA reduced its expression to basal level in human fibroblasts (Supplementary Fig. 5C). HDDC3 expression was not affected in all tested conditions (Supplementary Fig. 5D). These data suggest that UCP2 expression is directly regulated by NOX4 in human cells, which may explain a possible developmental compensatory mechanism in absence of Nox4 in mice. On the other hand, no obvious relationship between NOX4 and HDDC3 was observed in human cells.

### 3.5 Deletion of NOX4 has no impact on cellular metabolism of mouse skin fibroblast

**Fig. 6:**
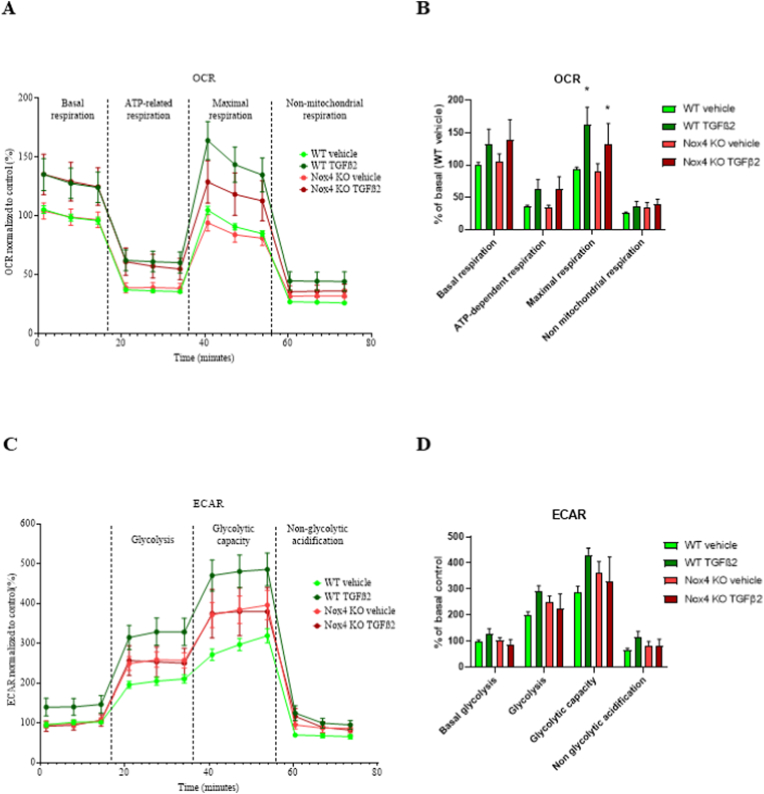
NOX4 deficiency did not affect cellular metabolism. A. Representation of the course of Seahorse XF Cell Mito Stress assay in WT and Nox4 KO primary skin fibroblasts in control conditions and after stimulation with TGF-β2 for 24h; B. Bar graphs representing the average of 3 time points for each measured phase during Seahorse XF Cell Mito Stress assay in WT and Nox4 KO primary skin fibroblasts in control conditions or after stimulation with TGF-β2 for 24h; C. Representation of the course of Seahorse XF Glycolysis Stress assay in WT and Nox4 KO primary skin fibroblasts in control conditions or after stimulation with TGF-β2 for 24h; D. Bar graphs representing the average of 3 time points for each measured phase during Seahorse XF Glycolysis Stress assay in WT and Nox4 KO primary skin fibroblasts in control conditions or after stimulation with TGF-β2 for 24h; WT Control (light green lines and bars) n = 8; WT TGF-β2 (dark green lines and bars) n = 8; Nox4 KO Control (red line and bars) n = 8; Nox4 KO TGF-β2 (dark red lines and bars) n = 8; OCR – oxygen consumption rate, ECAR – extracellular acidification rate; Data shown as mean ± SEM; ns – not significant, *– p < 0.05; 2way ANOVA. (For interpretation of the references to color in this figure legend, the reader is referred to the Web version of this article.)

To measure a potential impact of NOX4 on cellular metabolism, we measured mitochondrial activity and glycolysis using the Seahorse technology. In order to evaluate mitochondrial respiration of Nox4 KO fibroblasts, we performed a Seahorse XF Cell Mito Stress Test, which measures oxygen consumption in live cells following different pharmacological treatments, namely oligomycin, FCCP and a mixture of rotenone and antimycin A. Altogether, the pattern of OCR was typical of a Seahorse experiment [61] and showed that TGF-β2 treatment appeared to increase global OCR as previously described [62,63] although it was only significant different in maximal respiration conditions (Fig. 6A and B). Injection of oligomycin decreased basal OCR to non-significantly different levels in all investigated groups (Fig. 6A and B). FCCP injection, which reveals the maximal mitochondrial respiration, increased OCR in all tested group to levels similar or slightly higher to basal respiration (Fig. 6A and B). Maximal mitochondrial respiration was significantly higher in TGF-β2-treated conditions. However, this increase was similar in both WT and Nox4 KO groups, indicating that it was independent of Nox4. After rotenone and antimycin A mixture, the non-mitochondrial respiration diminished OCR in all tested groups to non-significantly different levels (Fig. 6A and B).

The Seahorse XF Cell Glycolysis Stress Test ECAR profile was typical of a Seahorse experiment [61]. Altogether, it appeared that TGF-β2 induced higher extracellular acidification in WT cells, however, this difference was not significant. Initial non-glycolytic capacity showed non-significantly different ECAR in all tested conditions (Fig. 6C and D). Induction of glycolysis with glucose injection revealed significant upregulation of glycolysis in all groups (Fig. 6C and D). Injection of oligomycin revealed similar remaining glycolytic capacity in all tested groups (Fig. 6C and D). Injection of 2-DG inhibited glycolysis in all tested conditions to similar level (Fig. 6C and D).

### 3.6 Deletion of NOX4 does not impact the mitochondrial quantity

**Supplementary Fig. 6:**
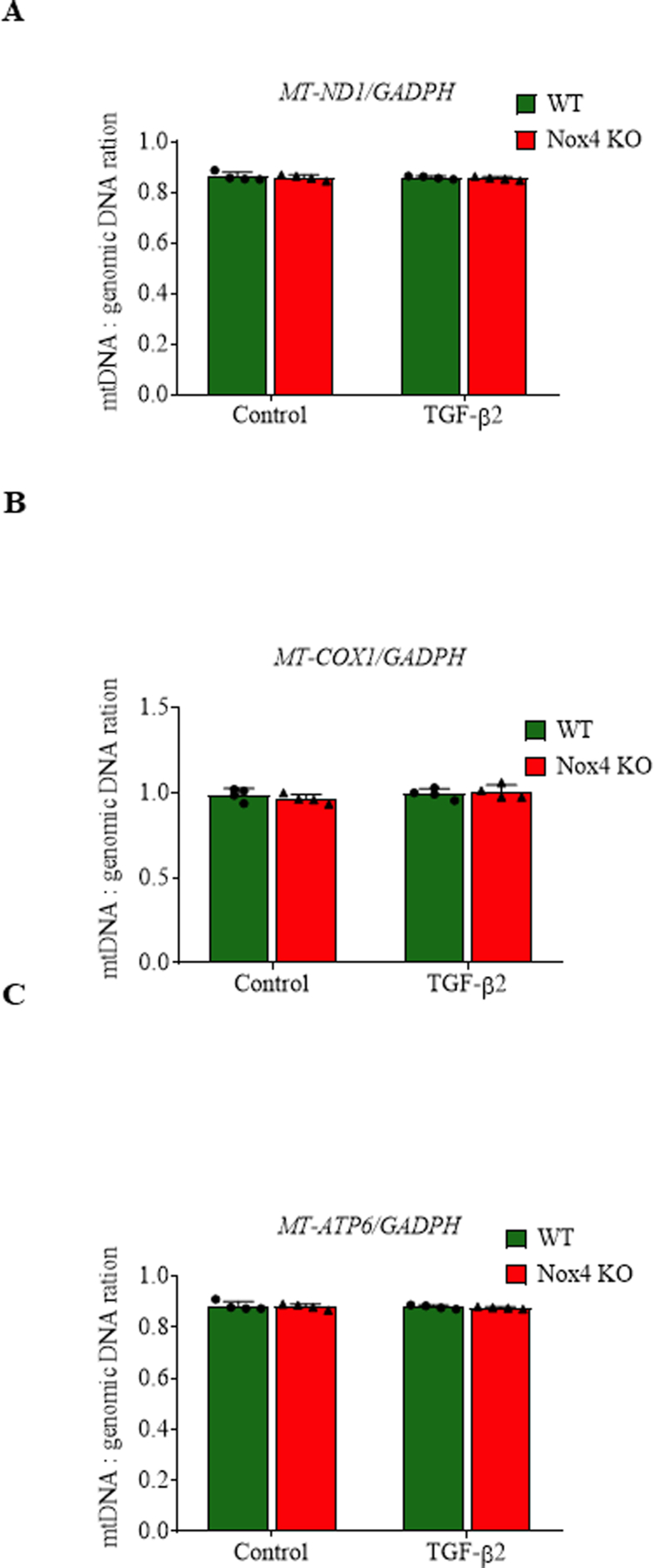
Mitochondrial content in Nox4-deficient primary mouse fibroblasts. A., B. and C. Ratio of mitochondrial DNA to genomic DNA defined as a ration of three chosen mitochondrial genes – MT-Nd1 (A), MT-Cox1 (B) and MT-Atp6 (C) to GADPH; WT (green bars) n = 4, Nox4 KO (red bars) n = 4; Data shown as mean ± SD; Two-way ANOVA.

In order to determine the possible impact of Nox4 on the amount of mitochondria, we determined the mitochondrial DNA content and compared it to genomic DNA content by qPCR. We did not find difference in the amount of mitochondrial DNA between WT and Nox4 KO fibroblasts without and with TGF-β2 (Supplementary Figs. 6A, B, C).

Altogether, we showed that Nox4 deletion does not interfere with physiological wound healing *in vivo* and TGF-β-mediated myofibroblasts differentiation *in vitro.* Nox4 deletion led to upregulation of *Ucp2* and *Hddc3* even without TGF-β2 and downregulation of *Islr* expression in myofibroblasts. However, changes in these genes does not affect either cellular metabolism or mitochondrial quantity.

## 4 Discussion

In this study, we aimed at elucidating the role of NOX4 during physiological wound healing and differentiation of skin fibroblasts into myofibroblasts. We found that NOX4 did not impact physiological wound healing in mouse. *In vitro*, NOX4 was strongly upregulated in mouse and human skin fibroblasts treated by TGF-β1 and TGF- β2, but NOX4 was dispensable for myofibroblast differentiation and cellular bioenergetics. However, we documented an upregulation of the redox modulators *Ucp2* and *Hddc3* in primary skin fibroblasts isolated from Nox4-deficient mice.

TGF-β is the main growth factor involved in myofibroblast differentiation. TGF-β induces massive gene expression changes, including typical proteins of the ECM and ⍺-SMA, leading to a typical contractile myofibroblast phenotype. During skin wound healing, fibroblasts proliferate and differentiate into myofibroblasts, inducing contraction of the wound. In our study, we confirmed that contraction of the wound was preponderant during wound closure in mouse and that Nox4 deletion had no impact on the specific myofibroblast differentiation markers ⍺-SMA and collagen during skin wound repair. A previous study had shown a slight, but significant delay in wound closure using the same Nox4 KO mouse strain [32]. However, in our study, the time necessary for wound closure was identical in WT and Nox4 KO mice. We have no clear answer for these contrasting results. Variation of normal microbiota present on the skin of this mouse strain may partly explain this discrepancy. Indeed Nox4-derived H_2_O_2_ may have a role in host defense or at least the regulation of microbial niche, similarly to NOX1 or DUOX in the gut [64,65] as well as NOX2 in macrophages and during the neutrophil oxidative burst [66].

There are three TGF-β isoforms in mammals [67]. TGF-β1 and TGF-β2 exhibit similar stimulating effect on wound healing and myofibroblasts differentiation [68], while TGF-β3 acts as their antagonist [69,70]. NOX4 was induced by TGF-β1 and TGF-β2 in both mouse and human skin fibroblasts to similar levels at identical concentrations, suggesting a common mechanism of NOX4 induction by these two TGFβ isoforms.

Normal wound healing shares multiple features of fibrosis, including TGF-β-mediated myofibroblast differentiation. Accordingly, *in vitro* TGF-β-induced myofibroblast formation is often used as a surrogate model to study the mechanisms governing fibrotic diseases.

In this context, several studies have described NOX4 as a key mediator of myofibroblast differentiation using cardiac [20], pulmonary [19], or intestinal fibroblasts [71], among others. Based on these studies, NOX4 inhibition has been proposed as an anti-fibrotic therapy and NOX4 inhibitors are currently evaluated in clinical trials for several fibrotic diseases [72]. In our study, NOX4 was not involved in skin fibroblast to myofibroblast differentiation. In particular, an unbiased RNAseq analysis showed that the typical molecular pathways induced by myofibroblast differentiation did not differ between WT and Nox4 KO skin mouse fibroblasts. We confirmed that NOX4 was not essential for myofibroblast differentiation using primary skin fibroblasts from p22^phox^-deficient mouse and human skin fibroblasts isolated from a patient carrying a p22^phox^ mutation and human skin fibroblasts treated with NOX4 siRNA. Intriguingly, pharmacological treatment with GKT137831 and DPI mitigated ⍺-SMA expression in skin fibroblasts treated with TGF-β1 independently of NOX4. DPI is a potent NOX inhibitor [73], however, DPI is not selective, as it potently inhibits other flavoprotein oxidases including nitric oxide synthase [74], xanthine oxidase [75], complex I of the mitochondrial chain [76], NADPH cytochrome P450 oxidoreductase (CYP450) [77] and all NOX isoforms [78]. GKT137831 (Setanaxib) is an experimental antifibrotic drug described as a NOX1/NOX4 inhibitor [79], which is currently under clinical evaluation. However, its mode of action and specificity are uncertain as it displays antioxidant properties similar to its close analogue GKT136901 [78,80]. The fact that DPI inhibited myofibroblast differentiation at low concentration (1 μM) suggests that other flavoenzymes are involved in this process. Although GKT137831 slightly mitigated NOX4 upregulation, it inhibited myofibroblast differentiation independently of NOX4. As GKT137831 is not known as a flavoenzyme inhibitor, this argues for another ROS generating system. In the human foreskin fibroblasts used in this study, NOX1 is absent, but DUOX1 and 2 are present (data not shown). DUOX1 may be the target for DPI and GKT137831-mediated mitigation of myofibroblast differentiation. Indeed, in a wound healing model in zebrafish larvae, a gradient of H_2_O_2_ produced by DUOX at the wound margin serves as signaling to help recruitment of leukocytes to the wound [81]. A role of DUOX1-derived H_2_O_2_ in myofibroblast differentiation was also proposed in TGF-β1-treated lung fibroblasts [82]. The RNAseq data did not point towards upregulation of another H_2_O_2_-generating flavoenzyme, which could compensate the deficiency of Nox4 as only 3 genes were differentially expressed in Nox4 KO fibroblasts treated with TGF-β1: an upregulation of *Ucp2* and *Hddc3*, and downregulation of *Islr* in *Nox4*-deficient skin fibroblasts in both basal and TGF-β2-treated conditions. *Islr* is ubiquitously expressed, but is enriched in fibroblasts. It is used to trace mesenchymal lineages [83] and its expression in cancer-associated fibroblasts (CAF) represents a favorable biomarker of cancer progression [84]. The *Hddc3* gene encodes Mesh1, a ubiquitous protein displaying a pro-oxidant activity as it acts as a NADPH phosphatase and depletes intracellular NADPH and glutathione and sensitizes cells to ferroptosis, a regulated form of cell death driven by the lethal accumulation of lipid hydroperoxides [32]. Ucp2 belongs to the family of inner mitochondrial membrane carrier proteins and to the subfamily of uncoupling proteins [34]. UCP2 is involved in numerous functions, such as cell proliferation [85,86], cell metabolism [87,88] and glycolysis [89]. UCP2 is regulated during cellular stress and the antioxidant response [90]. Although it appears counter-intuitive that the absence of a H_2_O_2_-generating system (i.e. NOX4) leads to an antioxidant response, a recent study showed that increased UCP2 in lung fibroblasts of idiopathic pulmonary fibrosis has a profibrotic impact associated with increased oxidative stress, impaired ATP synthesis and senescence [91]. In addition, in the context of NOX4, high levels of oxidative markers (8-hydroxyguanosine) and induction of the redox-sensitive nuclear factor erythroid 2-related factor 2 (Nfr2) transcription factor are described in the renal cortex of Nox4-deficient mice [92]. It is therefore possible that, in absence of NOX4, an elevated uncoupling by UCP2 leads to a mitochondrial compensatory ROS generation. However, mitochondrial function and glycolysis were not different in Nox4 KO fibroblasts with or without TGF-β2. We confirmed an increase of UCP2 following TGF-β1 in human fibroblasts, but NOX4 knock-down did not lead to further increased upregulation of UCP2 or HDDC3 in these cells. On the contrary, treatment with NOX4 siRNA or DPI decreased UCP2 expression to basal levels. The fact that *Ucp2* or *Hddc3* are upregulated in basal conditions (without TGF-β2) suggests a compensatory mechanism in the Nox4-deficient mouse.

Altogether, in spite of an extensive set of experiments, we did not observe an impact of NOX4 in wound closure, a minimal difference in molecular signature of primary fibroblasts from WT and Nox4 KO mice following TGF-β2 and no obvious differences in mitochondrial function and glycolysis. NOX4 upregulation during myofibroblast transition was neutral in our experimental conditions. There is no known post-translational activator of NOX4-dependent H_2_O_2_ generation as NOX4 activity is regulated at the transcriptional level [40]. Rozycki and collaborators [93] have proposed a two-hit mechanism for epithelial-mesenchymal transition (EMT) of kidney tubular cells: NOX4 induction by TGF-β alone is insufficient for EMT, which requires another pre-requisite, namely the disruption intercellular contacts, e.g. following a mechanical stimulus, which activate the cytoskeleton-regulated transcription factors MRTF and TAZ/YAP. Further studies may address if a similar mechanism leading cytoskeleton and NOX plays a role in fibroblast to myofibroblast transition. As of today, the physiological role of NOX4 is still not fully understood as no disease-causing mutations have been described in humans while Nox4 KO mice do not display an obvious phenotype even in kidney tubular cells where NOX4 is expressed at extremely high levels. Physiologically, NOX4 has been proposed to act as an O_2_ sensor [94] and to contribute in endothelial differentiation during development [95]. However, most of the work on NOX4 was done to study its role in pathological conditions. NOX4-derived H_2_O_2_ have been described to be a pathological mediator of fibrotic [19,96], cardiovascular [97] and neurological [98] diseases, but a protective effect of NOX4 has also been shown in hepatic cancer [99] or intestinal inflammation [100].

A key finding of this study is a so far unidentified link between NOX4 and the mitochondrial protein Ucp2 and the pro-oxidant protein Hddc3 in mouse fibroblasts. This unexpected aspect should be considered in the interpretation of data using Nox4-deficient mice and brings new insights for a potential role of NOX4 in mitochondria function and ferroptosis. Finally, we showed that the pharmacological compounds DPI and GKT137831 inhibited myofibroblast differentiation independently of NOX4 inhibition. Further studies aiming at identifying alternative enzymatic targets of DPI may bring new insights in the redox regulation of fibroblasts to myofibroblast differentiation.

## Authors contributions

A.M. S. performed the experiments; T. S. performed experiments with human fibroblasts; A. F. performed experiments with mouse kidney; Y. C. helped with the design, execution and analyzes; M-J. S. provided p22 mutated hDFs and hDFs; M-L. B-P. provided the α-SMA antibody and TGF-β2; D. A-L., B. P-C., A. M., A.M. S., K-H. K. designed the experiments,A.M. S and V. J. wrote the manuscript and prepared the figures; A. M. S. D-S. edited the manuscript.

## Funding

This study was granted by 10.13039/501100001711Swiss National Science Foundation to Brigitte Pittet-Cuénod and Ali Modarressi (Grant 310030–170132) and Karl-Heinz Krause (Grant 31003A-179478).

## Conflicts of interest

All authors have no competing interests to declare.

**Supplementary table 1:** Sequence of primers used for genotyping

1	*Nox4* WT sequence	TCA-TGA-CAG-TTG-GGG-ACA-AA	TTG-AAA-ATT-CAA-CAC-AAG-TCT-CC
2	*Nox4* KO sequence	TCA-TGA-CAG-TTG-GGG-ACA-AA	AAC-GTC-GTG-ACT-GGG-AAA-AC
3	*Cyba* WT sequence	CAA-GCC-TGC-CTG-AGT-TTG-G	CAA-ACT-AGG-CCA-GCT-GAA-GC
4	*Cyba* KO sequence	CGC-TCG-TGT-ATT-TCT-ACT-GTA-ACG	GAA-GCA-GCA-CAT-ATC-TGT-CAT-CC

**Supplementary Table 2:** Sequence of primers for qPCR.

1	*Cyba*	CTG-CTG-GAC-GTT-TCA-CAC-AG	AGA-GTA-GGC-GCC-GAA-ATA-CC
2	*Acta2/ACTA2*	GTC-CCA-GAC-ATC-AGG-GAG-TAA	TCG-GAT-ACT-TCA-GCG-TCA-GGA
33	*Nox4/NNox4/NOX4OX4*	GCT-CAT-TTC-CCA-CAG-ACC-TGGCT-CAT-TTC-CCA-CAG-ACC-TG	CTC-CGC-ACA-ATA-AAG-GCA-CACTC-CGC-ACA-ATA-AAG-GCA-CA
44	*Ucp2*	ATG-GTT-GGT-TTC-AAG-GCC-ACA	CGG-TAT-CCA-GAG-GGAA-AGT-GAT
5	*UCP2*	GGA-GGT-GGT-CGG-AGA-TAC-CAA	ACA-ATG-GCA-TTA-CGA-GCA-ACA-T
6	*Eef1*	TCC-ACT-TGG-TCG-CTT-TGC-T	CTT-CTT-GTC-CAC-AGC-TTT-GAT-GA
7	*EEF1*	AGC-AAA-AAT-GAC-CCA-CCA-ATGAGC-AAA-AAT-GAC-CCA-CCA-ATG	GGC-CTG-GAT-GGT-TCA-GGA-TAGGC-CTG-GAT-GGT-TCA-GGA-TA
88	*GadphGadph*	TCC-ATG-ACA-ACT-TTG-GCA-TTGTCC-ATG-ACA-ACT-TTG-GCA-TTG	CAG-TCT-TCT-GGG-TGG-CAG-TGACAG-TCT-TCT-GGG-TGG-CAG-TGA
9	*GADPH*	GCA-CAA-GAG-GAA-GAG-AGA-GAC-	AGG-GGA-GAT-TCA-GTG-TGG-TG
10	*Hddc3*	TGC-ACC-CCT-ACA-GGA-TGG-TC	AAG-CCC-TTT-TAC-CAC-CTG-GG
11	*HDDC3*	GCT-GCA-CCC-CAG-AGG-TAA-AA	CTG-GCA-AGC-TTG-GTT-CAA-GG
12	*P0*	AAT-CTC-CAG-AGG-CAC-CAT-TG	GTT-CAG-CAT-GTT-CAG-CAG-TG
13	*MT-Nd1*	TCA-CTA-TTC-GGA-GCT-TTA-CGA-GC	CAT-ATT-ATG-GCT-ATG-GGT-CAG-GC
14	*MT-Cox1*	GAC-TTG-CAA-CCC-TAC-ACG-GA	CCG-GTT-AGA-CCA-CCA-ACT-GT
15	*MT-Atp6*	GCA-GTC-CGG-CTT-ACA-GCT-AA	GGT-AGC-TGT-TGG-TGG-GCT-AA

## Data Availability

The complete list of significantly up- or downregulated genes in all conditions are documented in GEO database under the accession number GSE197562
